# Biogenesis and Proteolytic Processing of Lysosomal DNase II

**DOI:** 10.1371/journal.pone.0059148

**Published:** 2013-03-13

**Authors:** Susumu Ohkouchi, Masahiro Shibata, Mitsuho Sasaki, Masato Koike, Paul Safig, Christoph Peters, Shigekazu Nagata, Yasuo Uchiyama

**Affiliations:** 1 Department of Cell Biology and Neuroscience, Juntendo University School of Medicine, Tokyo, Japan; 2 Division of Gross Anatomy and Morphogenesis, Niigata University Graduate School of Medical and Dental Sciences, Niigata, Japan; 3 Unit of Molecular Cell Biology and Transgenic Research, Institute of Biochemistry, Christian Albrecht University Kiel, Kiel, Germany; 4 Institut für Molekulare Medizin und Zellforschung, Albert-Ludwigs-Universität Freiburg, Freiburg, Germany; 5 Department of Medical Chemistry, Graduate School of Medicine, Kyoto University, Kyoto, Japan; Universität Stuttgart, Germany

## Abstract

Deoxyribonuclease II (DNase II) is a key enzyme in the phagocytic digestion of DNA from apoptotic nuclei. To understand the molecular properties of DNase II, particularly the processing, we prepared a polyclonal antibody against carboxyl-terminal sequences of mouse DNase II. In the present study, partial purification of DNase II using Con A Sepharose enabled the detection of endogenous DNase II by Western blotting. It was interesting that two forms of endogenous DNase II were detected – a 30 kDa form and a 23 kDa form. Neither of those forms carried the expected molecular weight of 45 kDa. Subcellular fractionation showed that the 23 kDa and 30 kDa proteins were localized in lysosomes. The processing of DNase II *in vivo* was also greatly altered in the liver of mice lacking cathepsin L. DNase II that was extracellularly secreted from cells overexpressing DNase II was detected as a pro-form, which was activated under acidic conditions. These results indicate that DNase II is processed and activated in lysosomes, while cathepsin L is involved in the processing of the enzyme.

## Introduction

Apoptosis is cell death that results from a sequence of physiological processes that are triggered by pathological stimuli. A distinguishing feature of apoptotic cell death is genomic DNA fragmentation into oligonucleosomes [1]. The degradation of genomic DNA in dying cells (cell-autonomous degradation of DNA) is executed by caspase-activated DNase (CAD). Under normal conditions, CAD activity is suppressed by an inhibitor of CAD (ICAD). However, when cells undergo apoptosis, activated caspase-3 or -7 cleaves ICAD, which allows activation of CAD. The activated enzyme is translocated into nuclei where it cleaves genomic DNA into nucleosomal units that are responsible for the characteristic “DNA ladder” upon electrophoresis [2], [3].

Although CAD is indispensable for *in vivo* programmed cell death (PCD), transgenic mice with a functional CAD deficiency and CAD knockout mice both develop normally [4]–[6]. Terminal deoxynucleotidyl transferase-mediated dUTP-biotin nick-end labeling (TUNEL)-positive cells have been observed in CAD-deficient macrophages that phagocytose dying cells. Inhibition of lysosomal enzyme activity by treatment with chloroquine, which raises the pH in lysosomes [5], prevents degradation of apoptotic DNA in CAD-deficient macrophages. These lines of evidence indicate that a DNase other than CAD is present in the lysosomes of macrophages.

Until now, two lysosomal nucleases have been well characterized and their *in vivo* roles have been determined in mice lacking the proper enzymes [7], [8]. One of these enzymes is deoxyribonuclease II (DNase II, also called DNase IIα: DNase IIβ is expressed only in eye tissue). Deficiency of DNase II itself is not embryonic-lethal but mice deficient in DNase II (*DNase II^−/−^*) die during the later developmental stages [7], [9] because of constitutive interferon (IFN) β production [7], [10]. Moreover, mice that are doubly deficient in *DNase II* and *IFN-IR* (type-I interferon receptor) appear normal at birth, but gradually develop polyarthritis with age [11]. Macrophages in the embryos of *DNase II^−/−^* mice phagocytose, but cannot digest nuclei that are expelled from erythroid precursor cells. Undigested DNA can be observed in the spleen, liver and other tissues of the embryos [7]. An *in vitro* experiment showed that macrophages isolated from *DNase II^−/−^* mice can not degrade the DNA of phagocytosed apoptotic thymocytes [6]. Thus, DNase II is required for the degradation of apoptotic DNA by macrophages.

The endogenous DNase II protein has been purified from the lysosomal fraction, in which DNase II activity was recovered and activity of lysosomal cathepsin D and acid phosphatase was detected [12], [13]. Acid DNase activity was detected in various tissues in both mice and humans [14], [15], while the DNase II activity was detected under acidic conditions and independent of divalent cations [16]. Therefore, it is likely that DNase II is localized in lysosomes. At present, however, localization of DNase II in various animal tissue cells has not been well characterized using immunohistochemistry, although the role of the protein has been identified [17].

Reports on the biochemical properties of DNase II remain equivocal. Several different molecular weights that have been reported for human DNase II differ between the reported data. These have been listed as 45 kDa [18], [19] and 38 kDa [20] forms in human cell lines, and a 32 kDa protein in the liver and urine [21]. Purified porcine DNase II was determined by gel filtration to have a molecular weight of 45 kDa, but SDS-PAGE showed molecular weights of 35 and 10 kDa [22]. Although processing of porcine DNase II by proteases has been proposed [23], [24], human DNase II does not seem to undergo processing [18], [19].

To better understand the characteristics of DNase II, it is important to determine whether DNase II is localized in lysosomes and undergoes proteolytic processing. In the present study, we produced an anti-DNase II antibody for this purpose. Results of biochemical and immunohistochemical experiments to which this antibody was applied, indicated that DNase II is localized in the lysosomes of macrophages. Moreover, DNase II was processed when it was overexpressed in cell lines, and its processing was suppressed by protease inhibitors. We determined that DNase II also undergoes proteolytic processing *in vivo,* while its processing was dependent on cathepsin L.

## Materials and Methods

### Animals

The procedures involving animal care and sample preparation were approved by the Animal Experimental Committee of Juntendo University Graduate School of Medicine (Permit number: 240083) and performed in compliance with the regulations and guidelines for the care and use of laboratory animals of Juntendo University Graduate School of Medicine. *DNase II^+/−^* [7], *DNase II^+/−^ IFN-IR^−/−^* [11], *cathepsin B^+/−^* [25], *D^+/−^* [26], [27], *and L^+/−^* [28] mice were housed under specific pathogen-free conditions at Osaka University and at Juntendo University. The genotype of *DNase II^+/−^ IFN-IR^+/−^* mice was determined by PCR using genomic DNA with three primers (5′-GATTCGCAGCGCATCGCCTT-3′, 5′-CAGTGCCACAGAGGACCACT-3′, and 5′-GAGTCTTAGTCCTTTGCTCCG-3′) for the *DNase II* allele, and three additional primers (5′-ATTATTAAAAGAAAAGACGAGGCGAAGTGG-3′, 5′-AAGATGTGCTGTTCCCTTCCTCTGCTCTGA-3′, and 5′-CCTGCGTGCAATCCATCTTG-3′) for the *IFN-IR* allele. Genotyping for cathepsins B, D, and L mice was described previously [29]. Male C57BL/6J mice were purchased from Charles River.

### Cloning of DNase II cDNA and Plasmid Construction

Mouse DNase II (DNase II) cDNA was obtained by PCR using mouse spleen cDNA with a pair of primers (5′-CGGGATCCATGGCAACACTGAGATCGCTG-3′ and 5′-GGAATTCTCAGCTCCCCTCTATACAGGG-3′). A cDNA fragment of DNase II that corresponds to a carboxyl-terminal amino acid sequence of DNase II (DNase IIC: residues 237–353) attached with FLAG-His at the 3′-end for protecting the proteins from degradation during purification (data not shown) was ligated into pGEX6P-1 (GE Healthcare, Little Chalfont, Buckinghamshire, UK). In a similar manner, the same sequence of cDNA with a 3′-chitin-binding domain (CBD), which was from pTYB1 (NEB, Beverly, MA), was ligated into pMal-c2x (NEB, Beverly, MA). The plasmids were transformed into *E.coli* DH5α for expression of recombinant proteins. A plasmid vector that encodes the full-length DNase II with the carboxyl-terminal FLAG-His tag was constructed in a pcDNA3 vector for transfection into mammalian cells.

### Expression of Recombinant DNase II Protein in *E.coli* and Purification of the Protein

For the glutathione S-transferase (GST)-fused protein (GST-DNase IIC-FLAG-His), the *E.coli* transformed with the plasmid was cultured in 2×YT medium, and expression of the recombinant protein was induced by adding 0.5 mM IPTG in the culture at 16°C overnight. For maltose binding protein (MBP)-fused DNase II (MBP-DNase IIC-CBD), the *E.coli* were cultured in LB medium and expression of the protein was induced at 37°C for 3 h in the presence of 0.3 mM IPTG. GST-DNase IIC-FLAG-His was purified with Glutathione Sepharose, and MBP-DNase IIC-CBD was purified with amylose resin according to the manufacturer’s instructions.

### Generation of a Polyclonal Antibody against DNase II

An anti-DNase II polyclonal antibody was prepared by immunizing rabbits with GST-DNase IIC-FLAG-His. The antibody was purified with HiTrap NHS-activated HP (GE Healthcare, Little Chalfont, Buckinghamshire, UK) and immobilized with MBP-DNase IIC-CBD.

### Partial Purification of Endogenous DNase II from the Spleen Using Con A Sepharose

Immediately after livers and spleens were excised from *DNase II^−/−^ IFN-IR^−/−^* mice and those from *DNase II^+/+^ IFN-IR^−/−^*littermates, they were frozen in liquid nitrogen and stored at −80°C until use. The spleens were homogenized using a Polytron homogenizer in 1 ml of a lysis buffer (50 mM Tris-HCl (pH 7.5), 0.15 M NaCl) that contained a protease inhibitor cocktail (Nacalai Tesque, Kyoto, Japan). The lysates were centrifuged at 20,000×*g* for 10 min and the supernatant was applied to Con A Sepharose (GE Healthcare, Little Chalfont, Buckinghamshire, UK). After washing with a wash buffer (50 mM Tris-HCl pH 7.5, 0.5 M NaCl), bound proteins were eluted sequentially with 1 ml each of the wash buffer containing 0.1, 0.2, 0.3, 0.4 and 0.5 M α-methyl-D-mannoside. A small amount (10 µl) of each fraction was used for the assay of DNase II activity (see below). Residual fractions eluted (approximately 1 ml each) were precipitated by the addition of 10% trichloroacetic acid followed by incubation on ice for 1 h. After centrifugation at 20,000×*g* for 15 min and washing with 95% acetone, the precipitate was dissolved in 20 µl of SDS-sample buffer (63.5 mM Tris-HCl (pH 6.8), 5% glycerol, 2% SDS, and 2% 2-mercaptoethanol). For Western blotting, 5 µl of each precipitated fraction were used.

### DNase II Assay

Activity of DNase II was analyzed according to the method described previously [30] with a slight modification. Briefly, 1 µg of plasmid DNA (pBluescript) was incubated at 37°C for 2 h with 10 µl of Con A Sepharose eluted fractions in 100 µl of 50 mM acetate and 10 mM EDTA (pH 4.7). The reaction was stopped by the addition of an equal volume of 1 M Tris-HCl (pH 8.0). After phenol/chloroform extraction and ethanol precipitation, the sample was dissolved in TE buffer and half of the sample was subjected to electrophoresis on a 1% agarose gel.

### Expression of DNase II in 293FT Cells and Western Blotting

Plasmid DNA (4 µg) was transiently transfected into 293FT cells (Invitrogen, Carlsbad, CA) using LipofectAmine 2000 (Invitrogen, Carlsbad, CA) reagent according to the manufacturer’s instructions. Mock transfection was performed as a negative control experiment. Two days after transfection, the cells were harvested, washed, lysed, and centrifuged. The resultant supernatant was used as the cell lysate. The protein concentration of the lysate was determined using a BCA protein assay kit (Pierce, Rockford, IL). The samples (3 µg each) were subjected to SDS-PAGE followed by Western blotting onto a PVDF membrane (Immobilon-P; Millipore, Billerica, MA) [31]. Antibodies against DNase II (8 µg/ml) or FLAG-tag (Sigma-Aldrich, St. Louis, MO) were used as the primary antibodies.

### Subcellular Fractionation

COS-1 cells [32] that stably expressed DNase II-FLAG-His were harvested, washed, and resuspended in 1 ml of buffer A (10 mM Tris-HCl, 0.25 M sucrose, pH 7.5) containing a proteinase inhibitor cocktail. The cells were lysed by forced passage through a 25-gauge needle. The lysates were centrifuged at 1,000×*g*. A small amount of the postnuclear supernatant (PNS) was used for Western blotting. The residual PNS fraction was centrifuged at 11,000×*g* for 20 min to obtain pellet (P2) and supernatant (S2) fractions. The P2 was resuspended in buffer A, layered onto 28% Percoll, then ultracentrifuged at 97,000×*g* for 40 min using a Beckman SW41Ti rotor. After removal of the Percoll, the bottom layer (lysosomal fraction) was resuspended in buffer A and lysed by the addition of SDS to 0.1%. The S2 fraction was ultracentrifuged at 97,000×*g* for 1 h using the same rotor. The pellet (microsomal fraction) was resuspended in buffer A and lysed by the addition of SDS to 0.1%. The amount of protein in the lysed PNS, the lysosomal and microsomal fraction, was determined using a BCA assay and 2 µg of each fraction was used for Western blotting with antibodies against cathepsin D [29], Bip [33], FLAG, and DNase II.

### Processing of DNase II

COS-1 cells that stably expressed DNase II-FLAG-His were cultured in DMEM, containing a proteinase inhibitor cocktail (×200), and either E-64d (10 µg/ml), pepstatin A (6 µM), or DMSO (control) for 24 h. The cells were then harvested, lysed, and analyzed by Western blotting. The spleen and liver from cathepsin B, L (8 weeks), or D (postnatal day 23)-deficient mice were lysed and each lysate was applied to Con A Sepharose as described above, with the exception of bound materials, which were eluted with a wash buffer containing 0.5 M α-methyl-D-mannoside. Proteins were quantified by measurement of the density of each protein band, using an Image Gauge software program (Fuji Photo Film, Japan). The ratios of the amounts of the 23-kDa to 30-kDa proteins and the means ± standard deviation were calculated for three independent experiments.

### Partial Purification and Immunoprecipitation of Extracellular DNase II

COS-1 cells or COS-1 cells that stably expressed DNase II-FLAG-His were cultured in DMEM, containing 0.5 mM mannose-6-phosphate, and/or a protease inhibitor cocktail (×200) for 2 days. The culture media were harvested, filtered with a 0.22 µm filter (MILLEX GP; Millipore, Billerica, MA), and added by the proteinase inhibitor cocktail (×100). Extracellular DNase II was partially purified by using Con A Sepharose, and 0.5 M α-methyl-D-mannoside-eluted fractions were then subjected to immunoprecipitation using an anti-FLAG M2 antibody. The extracellular DNase II was further eluted in the immunoprecipitated solution with 200 µg/ml FLAG-peptide. The Con A and anti-FLAG-immunoprecipitated (IP) fractions were subjected to both Western blot analysis and DNase II assay.

### Extracellular DNase II Activity Under Acidic Conditions

To examine extracellular DNase II activity, 100 µl of a reaction solution was prepared that contained 10 µl of the Con A or anti-FLAG IP fraction from COS-1 cells that stably expressed DNase II-FLAG-His, and 50 mM acetate buffer (pH4.7) with 10 mM EDTA. Two µg of plasmid DNA was dissolved in the solution and incubated at 37°C for 2 h. DNA fragments in the remainder of the reaction mixture (60 µl), to which an equal volume of 1 M Tris-HCl (pH 8.0) was added, were extracted with phenol/chloroform and precipitated with ethanol. The obtained DNA fragments were dissolved in TE buffer and half of them were subjected to electrophoresis on a 1% agarose gel. For pre-incubation assay, 100 µl of the reaction solution containing 10 µl of the Con A fraction or anti-FLAG IP fraction from COS-1 cells that stably expressed DNase II-FLAG-His, 50 mM acetate buffer at pH 4.7, or 50 mM Tris-HCl buffer at pH 6.0 or 6.5, 10 mM EDTA, and proteinase inhibitor cocktail was pre-incubated at 37°C for 2 h. After pre-incubation, each reaction solution was adjusted to pH 6.0 or 6.5, and contained 200 mM Tris-HCl buffer and 10 mM EDTA with proteinase inhibitor cocktail (×100). Then, 2 µg of plasmid DNA was dissolved and incubated in the reaction solutions at 37°C for 3 h. After incubation, DNA fragments were purified and analyzed, as described above.

### Immunohisto/cytochemistry

Adult *DNase II^+/−^ IFN-IR^−/−^*, *DNase II^−/−^ IFN-IR^−/−^* and wild-type (wt) (C57BL/6J) mice (8 weeks of age) were deeply anesthetized with pentobarbital (25 mg/kg i.p.) and fixed by cardiac perfusion with 4% paraformaldehyde buffered with 0.1 M phosphate buffer (pH 7.2) (PB) containing 4% sucrose for light microscopy and with 4% paraformaldehyde-0.1% glutaraldehyde buffered with 0.1 M PB for immunoelectron microscopy.

For light microscopic immunohistochemistry, various tissues including spleen and bone (femur) were excised from the mice and further immersed in the same fixative for 2 h. Bones were decalcified in 4% sucrose-containing 5% EDTA for 10 days. Samples for cryosections were embedded in O.C.T. compound (Miles, Elkhart, IN) after cryoprotection with 15 and 30% sucrose solutions and cut into 10-µm sections with a cryostat (CM3050; Leica, Nussloch, Germany). The sections were placed on silane-coated glass slides and immunostained as described previously [27], [29] with anti-DNase II antibody (8 µg/ml). For double or triple immunofluorescent staining, cryosections were incubated with a mixture of rabbit anti-DNase II (1∶100), goat anti-mouse cathepsin L (1∶100) (R&D Systems, Minneapolis, MN), and rat anti-mouse lamp-1 (1∶100) (the Developmental Studies Hybridoma Bank, Iowa city, IA) or anti-DNase II with rat anti-CD68 (1∶100) (Serotec, Oxford, UK) at 4°C overnight, followed by incubation with a mixture of donkey, anti-rabbit and anti-goat, and anti-rat IgG coupled with Alexa Fluor 488 and 594 (Invitrogen, Carlsbad, CA), and Cy5 (Jackson Laboratory, Bar Harbor, ME), respectively, for 1 h at RT. The sections were then viewed under a confocal laser-scanning microscope (FV1000, Olympus, Tokyo, Japan).

The spleens of wt mice were excised and ultrathin cryosections were prepared as reported previously [27]. The sections were rinsed with PBS containing 0.02 M glycine, treated with 1% BSA in PBS for blocking, and were then incubated overnight at 4°C with rabbit anti-DNase II (1∶10) and rat anti mouse lamp-1 (1∶10) or CD68 (1∶10). The sections were then incubated for 1 h at room temperature with goat anti-rabbit and rat IgG conjugated with 5 and 10 nm colloidal gold particles, respectively (GE Healthcare and British Biocell International, respectively). The sections were observed with a Hitachi H-7100 electron microscope. For control experiments, ultrathin sections were directly incubated with the second antibody without pretreatment with the first antibody.

## Results

### Antibody Characterization by Western Blotting

**Figure 1:**
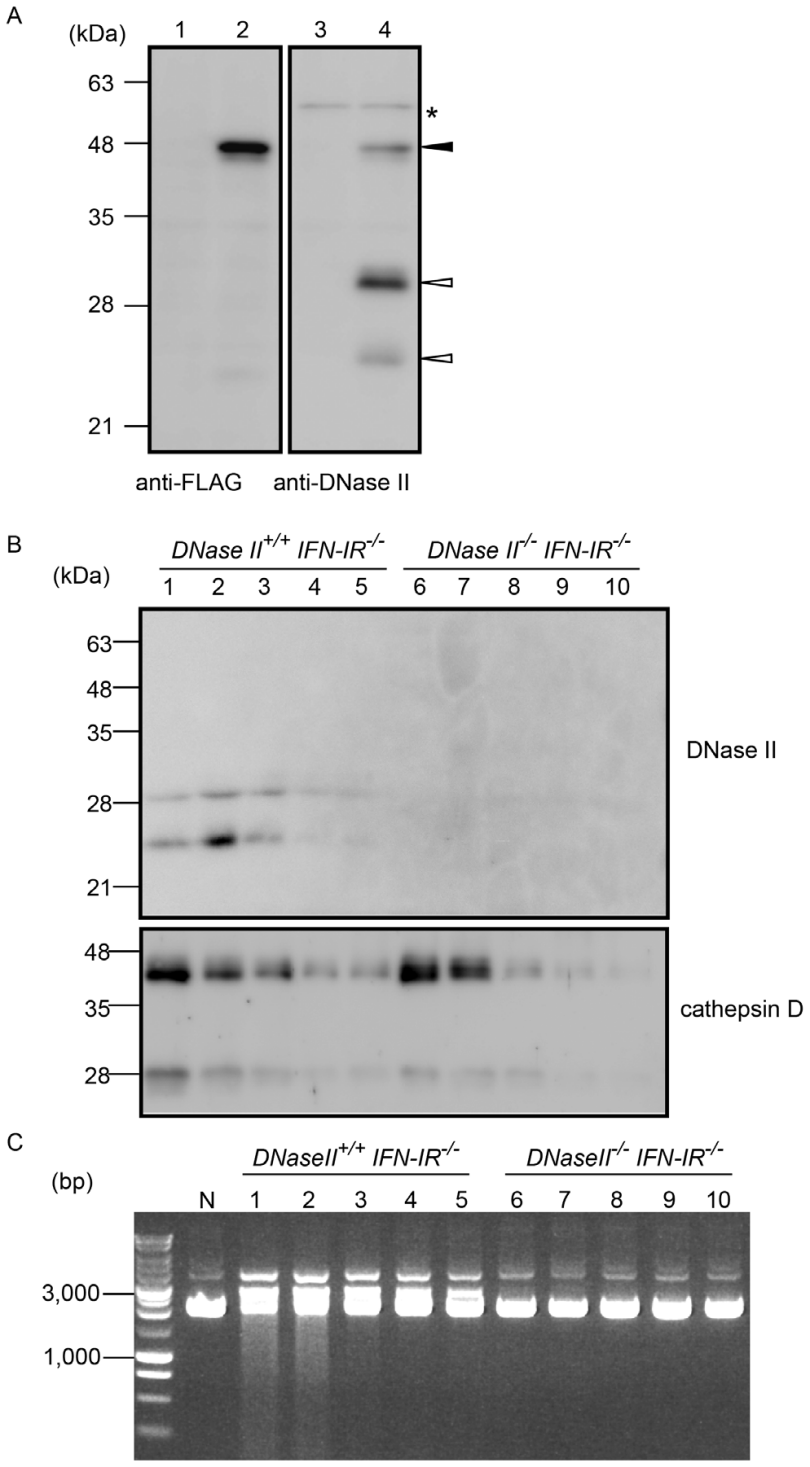
Anti-DNase II polyclonal antibody detects recombinant and endogenous DNase II proteins. A, Mock transfected (lane 1, 3) or transiently transfected (lane 2, 4) 293FT cells with a plasmid for expression of the mouse DNase II protein with a FLAG-His tag at the carboxyl-terminus. The cell lysates were analyzed by Western blotting using anti-FLAG M2 antibody (lanes 1, 2) or anti-DNase II antibody (lanes 3, 4). Closed arrowhead; DNase II-FLAG-His protein detected by both antibodies, open arrowheads; DNase II-FLAG-His protein detected only by anti-DNase II antibody. Asterisk indicates non-specific detection of protein bands. B, Analyses of Con A-eluted fractions. Spleen lysates from *DNase II^+/+^ IFN-IR^−/−^* mice and *DNase II^−/−^ IFN-IR^−/−^* mice were partially purified with Con A Sepharose. Fractions eluted with 0.1 (lanes 1, 6), 0.2 (lanes 2, 7), 0.3 (lanes 3, 8), 0.4 (lanes 4, 9), and 0.5 M (lanes 5, 10) α-methyl-D-mannoside were concentrated using a trichloroacetic acid precipitation, and the samples were analyzed by Western blotting using either the anti-DNase II antibody (upper panel), or anti-cathepsin D antibody (lower panel). Cathepsin D was used to confirm that a lysosomal glycosylated protein was extracted in the lysates and purified by Con A Sepharose. C, DNase activity of the eluted fractions. The eluted fractions from Con A Sepharose were directly assayed for DNase activity as described in the experimental procedures. N: negative control experiment without lysates.

To investigate the distribution of the DNase II protein, antiserum against the carboxyl-terminal sequence (amino acids 237–353) of mouse DNase II was prepared. The polyclonal antibody was purified by affinity chromatography with MBP-fused DNase II protein, and was tested in a Western blot of 293FT cells expressing DNase II-FLAG-His. The DNase II-FLAG-His protein was detected by the anti-FLAG antibody as a band with a molecular mass of approximately 45 kDa (Figure 1A lane 2, closed arrowhead). The absence of the band in the lysates of mock-transfected cells verified that the band was derived from an exogenously expressed protein (Figure 1A, lane 1). Interestingly, the polyclonal antibody against DNase II recognized three major protein bands, the largest one of which had the same molecular mass as that of the band detected by the anti-FLAG-antibody (Figure 1A, lane 4, closed arrowhead). The two other protein bands, which were recognized by the anti-DNase II antibody but not by the anti-FLAG antibody, had molecular masses of approximately 30 kDa and 23 kDa (Figure 1A, lane 4, open arrowheads). Next, we attempted to detect endogenous DNase II in the spleen lysates. However, no clear-cut difference was detected between the spleen samples from *DNase II^+/+^ IFN-IR^−/−^* and *DNase II^−/−^ IFN-IR^−/−^* mice when stained with anti-DNase II (data not shown). This result indicates that detection of endogenous DNase II is difficult, or even impossible, when normal lysates are used. Therefore, we performed partial purification of the DNase II protein from the splenic lysates using Con A Sepharose, which has been used previously for the purification of endogenous DNase II [14]. Con A-purified fractions that had been concentrated by trichloroacetic acid precipitation were analyzed by Western blotting. Since cathepsin D is a lysosomal glycosylated protein, it was detected in lysosomal fractions from both samples at this purification step (Figure 1B, lower panel). When the same samples were analyzed using the anti-DNase II antibody, two protein bands with molecular masses of 30 kDa and 23 kDa were observed in the fractions from *DNase II^+/+^IFN-IR^−/−^* mice but not in those from *DNase II^−/−^ IFN-IR^−/−^* mice (Figure 1B, upper panel). No 45-kDa bands were detected by the Western blot analyses of semi-purified samples. DNase II activity was simultaneously measured in these partially purified samples. Activity was detected only in the lysosomal fractions, in which the DNase II proteins were found by Western blotting, but not in DNase II-deficient fractions (Figure 1C). These results indicate that the immunoreactive bands correspond specifically to the endogenous DNase II proteins.

### Expression of DNase II in Macrophages from Mouse Spleen and Bone Marrow

**Figure 2:**
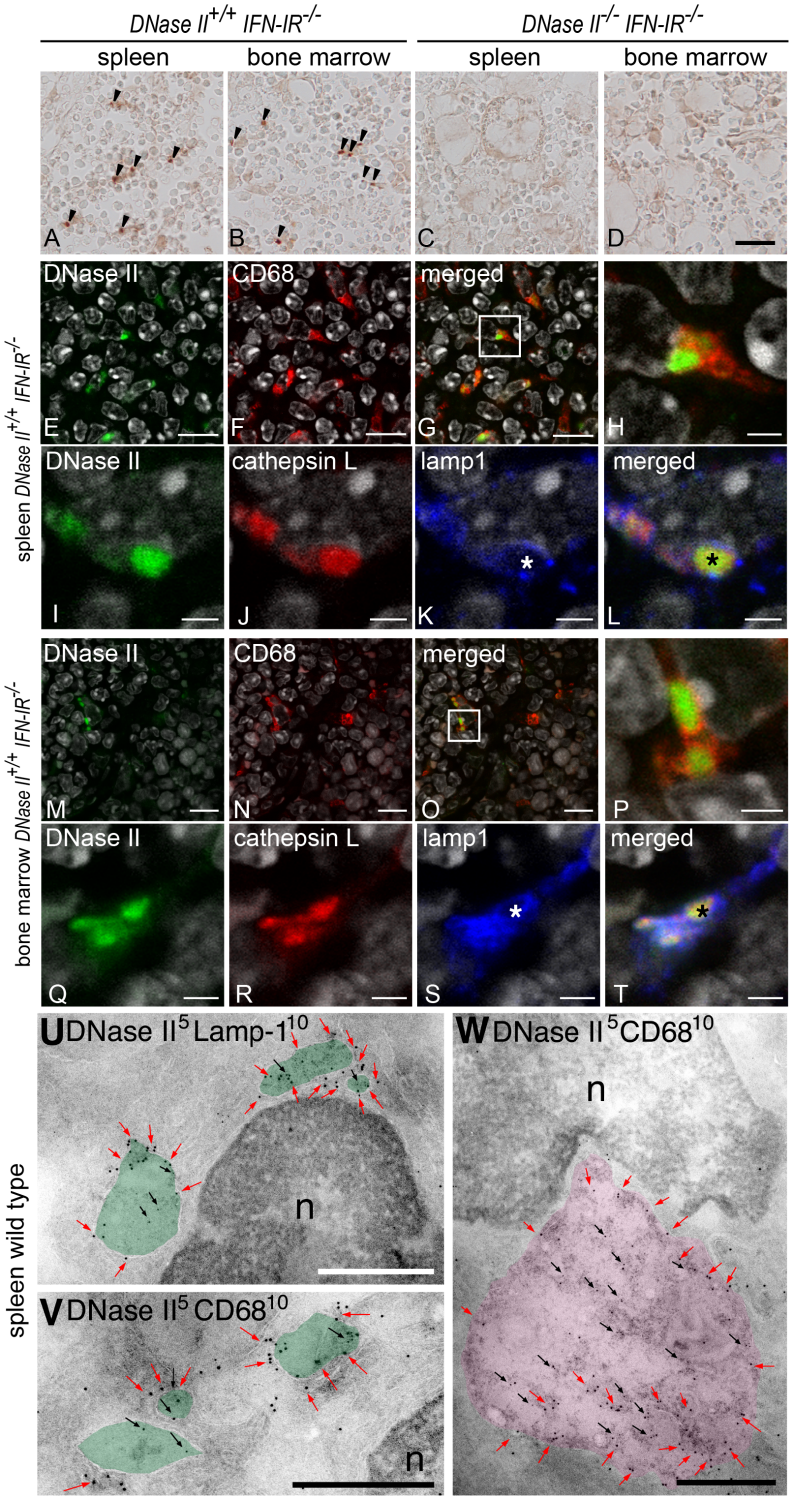
Immunohistochemical detection of DNase II. The spleen (A, C) and bone marrow of the femur (B, D) from *DNaseII^+/+^IFN-IR^−/−^* mice (A, B) and *DNaseII^−/−^ IFN-IR^−/−^* mice (C, D) were stained with anti-DNase II antibody. In the spleen, immunoreactivity for DNase II appeared as granules in the red pulp (A, arrowheads). Similar granular staining was detected in the bone marrow (B, arrowhead). No such granular staining was found in the spleen or femur of *DNaseII^−/−^ IFN-IR^−/−^* mice (C, D). Cryosections of the spleen (E–L) and bone marrow of the femur (M–T) from wt mice were stained with anti-DNase II (green) and anti-CD68 (red) (E–H and M–P) or with anti-DNase II (green), anti-cathepsin L (red), and anti-lamp1 (blue) (I–L and Q–T). Panels H and P are high magnification images of the insets of panels G and O, respectively. Nuclei were stained by DAPI (gray). Asterisks in K, L, S, T indicate lumen of heterophagolysosomes. Double-immunogold labeling of DNase II (5 nm) and lamp-1 (10 nm) (U) or CD68 (10 nm) (V, W) in mouse spleen. Positive signals indicating DNase II (black arrows) were localized in lamp1- (red arrows) or CD68- (red arrows) positive lysosomes (light green overlay) (U, V). DNase II was detected in abundant amounts in relatively larger CD68-positive heterophagolysosomes (light pink overlay) (W). N: nucleus. Bars: A–D = 20 µm; E–G and M–O = 10 µm; H–L and P–T = 2 µm; U–W = 500 nm.

DNase II is expected in the spleen and bone marrow since a previous report showed that undigested DNA is present in the macrophages of these tissues in *DNase II^−/−^ IFN-IR^−/−^* mice [11]. Therefore, the expression of the DNase II protein in these tissues was investigated using immunohistochemistry. In the spleen, immunoreactivity for DNase II was found to be granular and localized in the red pulp, where macrophages were abundant (Figure 2A arrowheads). On the other hand, DNase II immunoreactivity was only detected in certain types of bone marrow cells (Figure 2B). The positive signals of DNase II were specific, since the immunoreactivity was not observed in the cryosections of spleen and bone marrow obtained from *DNase II^−/−^ IFN-IR^−/−^* mice (Figure 2C and D).

Expression of the DNase II protein in spleen and bone marrow tissues was further investigated by double or triple immunofluorescent staining, using antibodies against CD68, a marker for macrophages [34], lamp1, a lysosomal membrane protein, and cathepsin L, a lysosomal cysteine proteinase to determine whether positive signals for DNase II are localized in the lysosomes and heterophagolysosomes of macrophages. The granular staining of DNase II was colocalized in CD68-positive macrophages and mononuclear cells (Figure 2E–H and M–P). By triple immunostaining, positive signals for DNase II were found to be colocalized with those for cathepsin L, and were also colocalized or surrounded by immunostaining for lamp1 (Figure 2I–L and Q–T). These results indicate that DNase II is localized in the lysosomes and heterophagolysosomes of macrophages/mononuclear cells.

Moreover, immunoelectron microscopy of ultrathin cryosections revealed that gold particles indicating the presence of DNase II were localized in lamp1- (light green overlay in Figure 2U) or CD68-positive lysosomes (light green overlay in Figure 2V). Remarkably, DNase II-positive signals were abundant in relatively larger (approximately 1 µm in diameter) CD68-positive heterophagolysosomes (light pink overlay in Figure 2W), which were consistent with the intense immunofluorescent signals for DNase II (Figure 2I–L).

### 30- and 23-kDa Forms of DNase II are Localized to Lysosomes

**Figure 3:**
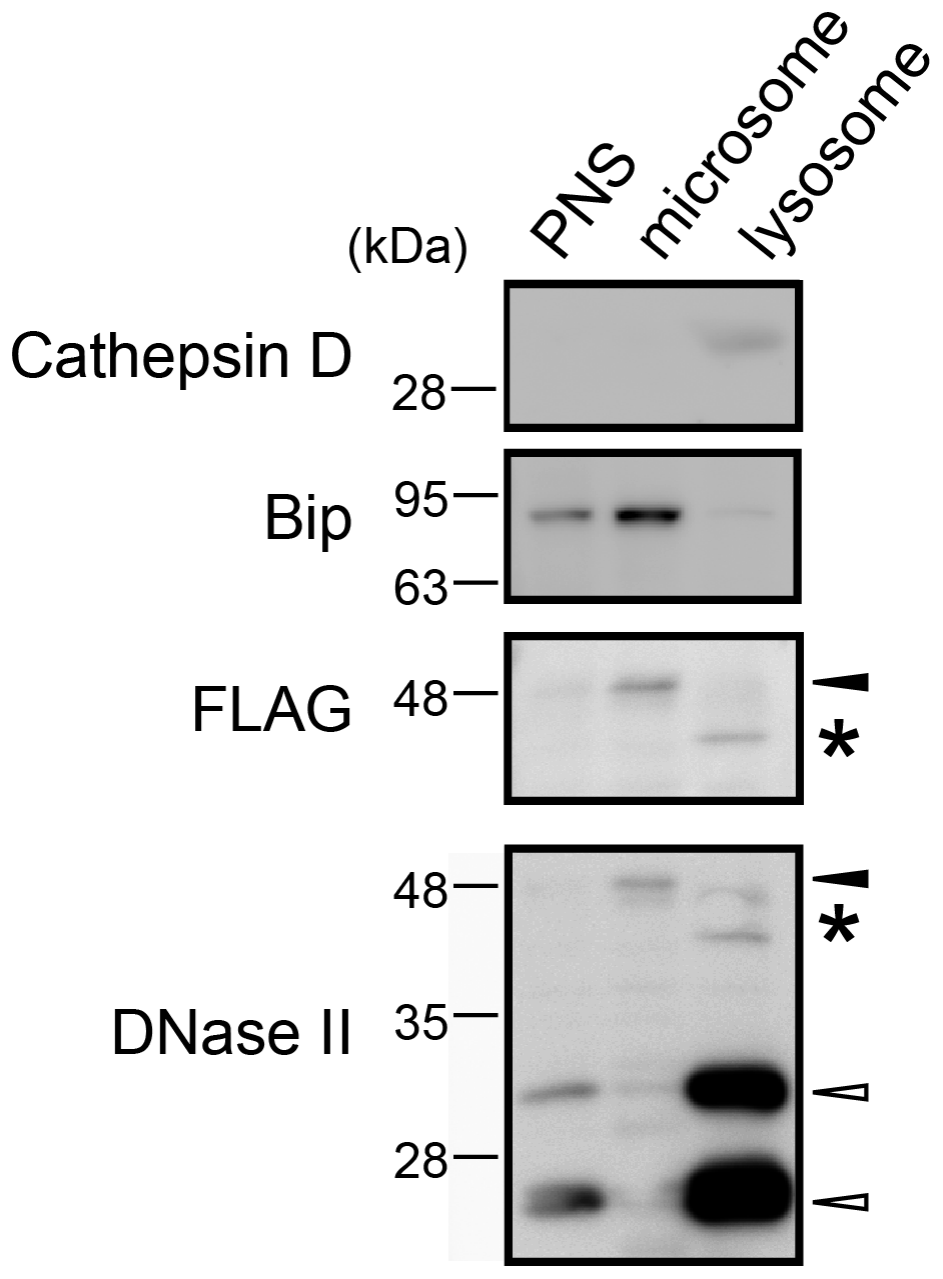
Subcellular fractionation of DNase II overexpressed in COS-1 cells. Subcellular fractionation of COS-1 cells stably overexpressing DNase II-FLAG-His was performed as described in the experimental procedures. Two µg of the samples from PNS, a lysosomal fraction and a microsomal fraction, were subjected to SDS-PAGE followed by Western blotting. Cathepsin D and Bip were used as markers of the lysosome and microsome (endoplasmic reticulum), respectively. Closed arrowhead; 45 kDa of proDNase II-FLAG-His protein, open arrowheads; 30 kDa and 23 kDa of processed forms of DNase II-FLAG-His proteins. Asterisk indicates non-specific detection of protein bands.

Subcellular fractionation using COS1 cells expressing DNase II-FLAG-His was performed to determine which form(s) of DNase II is present in lysosomes. The anti-FLAG antibody detected an immunoreactive band for the 45-kDa form of DNase II in the microsomal fraction, which contained an ER marker protein, Bip (Figure 3, closed arrowheads). By contrast, when the lysosomal fractions enriched in cathepsin D were analyzed with anti-DNase II antibody, two bands with strong immunoreactivity appeared at molecular masses of 30 and 23 kDa (Figure 3, open arrowheads). These results indicate that the 45-kDa form of DNase II is synthesized in the ER and processed during transport to the lysosomes via the Golgi apparatus.

### Effect of a Proteinase Inhibitor and a Deficiency of Lysosomal Proteinases during the Processing of DNase II in Lysosomes

**Figure 4:**
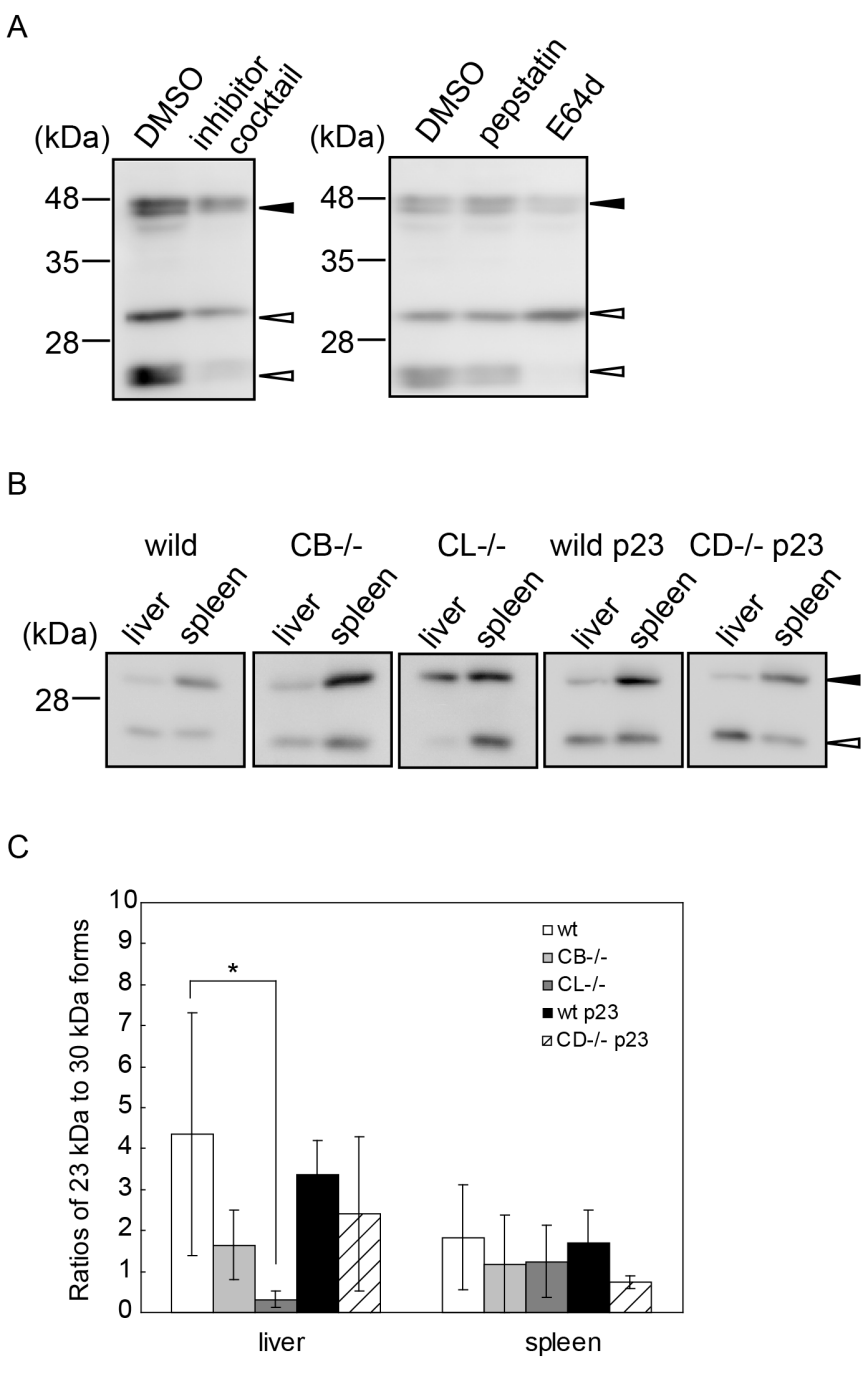
Processing of 30-kDa to 23-kDa DNase II proteins in lysosomes. A, Effect of proteinase inhibitors on processing of 30-kDa to 23-kDa DNase II proteins. COS-1 cells stably expressing DNase II-FLAG-His were cultured in the presence of a proteinase inhibitor cocktail, pepstatin A or E-64d. Closed arrowhead; proDNase II protein, open arrowheads; processed forms of DNase II proteins. B, C, Processing of 30 kDa DNase II in lysosomes was altered in cathepsin L-deficient mice. The liver and spleen from each mouse were applied to Con A Sepharose and analyzed by Western blotting as described in the materials and methods. Closed arrowhead; 30 kDa of processed form of DNase II protein, open arrowheads; 23 kDa of processed form of DNase II protein. C, Ratios of the amount (density) of the 23-kDa protein to that of the 30-kDa protein in the liver and spleen obtained from wild-type (wt), CB−/− and CL−/− mice, and from a wt mouse at postnatal day 23 (p23) and a CD−/− mouse at p23. Results (n = 3) are recorded as the mean ± S.D.; *P<0.05 versus wild type in Student’s *t*-test.

We noticed that the amount of the 23 kDa form of DNase II was significantly decreased when COS-1 cells expressing DNase II-FLAG-His were cultured in the presence of the proteinase inhibitor cocktail. A similar result was obtained when the cells were cultured in the presence of E-64d, an inhibitor of cysteine proteinases such as cathepsins B and L, but not in the presence of pepstatin A, an inhibitor of aspartic proteinases such as cathepsin D (Figure 4A). This finding indicates that the presence of an inhibitor of cysteine proteinases in the culture medium affected the processing of the 30 kDa DNase II. To investigate which lysosomal proteinases are involved in the *in vivo* processing of DNase II, the enzyme was partially purified with Con A Sepharose and used to detect the endogenous protein in tissues from cathepsin B-, L-, or D-deficient mice. Since expression of the DNase II protein was very low in vivo, no bands that were immunoreactive to the anti-DNase II antibody were detected in liver and spleen samples without partial purification by Con A Sepharose. Moreover, as shown in Figure 3, the presence of the proform of DNase II was very low even in COS-1 cells expressing DNase II-FLAG-His, compared to that of the 30-kDa and 23-kDa forms. It was hard to obtain a band that was immunoreactive to the anti-DNase II antibody around a molecular mass of 45 kDa in the liver and spleen samples after Con A Sepharose. This indicates that since expression of the pro-DNase II is very low (beneath detection limit) or the processing of pro-DNase II promptly occurs during transport from the ER to the Golgi apparatus *in vivo*, the pro-DNase II protein is hard to detect in the tissues of these mice. No clear-cut difference was detected in endogenous DNase II between partially purified samples from cathepsin D-deficient and wt mice, which was consistent with the result of the pepstatin A experiment (Figure 4B). As shown in Figure 3, both 30-kDa and 23-kDa forms of DNase II were localized in the lysosomal fraction and the antibody was raised against a carboxyl portion of DNase II (residues 237–353; see Materials and Methods). This indicates that 30-kDa and 23-kDa proteins are single-chain and heavy-chain forms of DNase II, respectively. To define the processing of DNase II in lysosomes, the ratio of the amount of the 23 kDa DNase II protein to that of the 30 kDa DNase II protein was examined in the liver and spleen of cathepsins B- and L-deficient mice, respectively by measuring the densities of both the 23 and 30 kDa proteins. The result showed that processing of the 30 kDa DNase II in the liver of cathepsin L-, but not cathepsin B-deficient mice was largely suppressed, compared with wt mice. By contrast, no difference in the processing of both 23 kDa and 30 kDa DNase II in cathepsin B- or L-deficient mice was observed in the spleen (Figure 4B and 4C).

### A Proform of DNase II that is Extracellularly Secreted from the Cells is Activated Under Acidic Conditions

**Figure 5:**
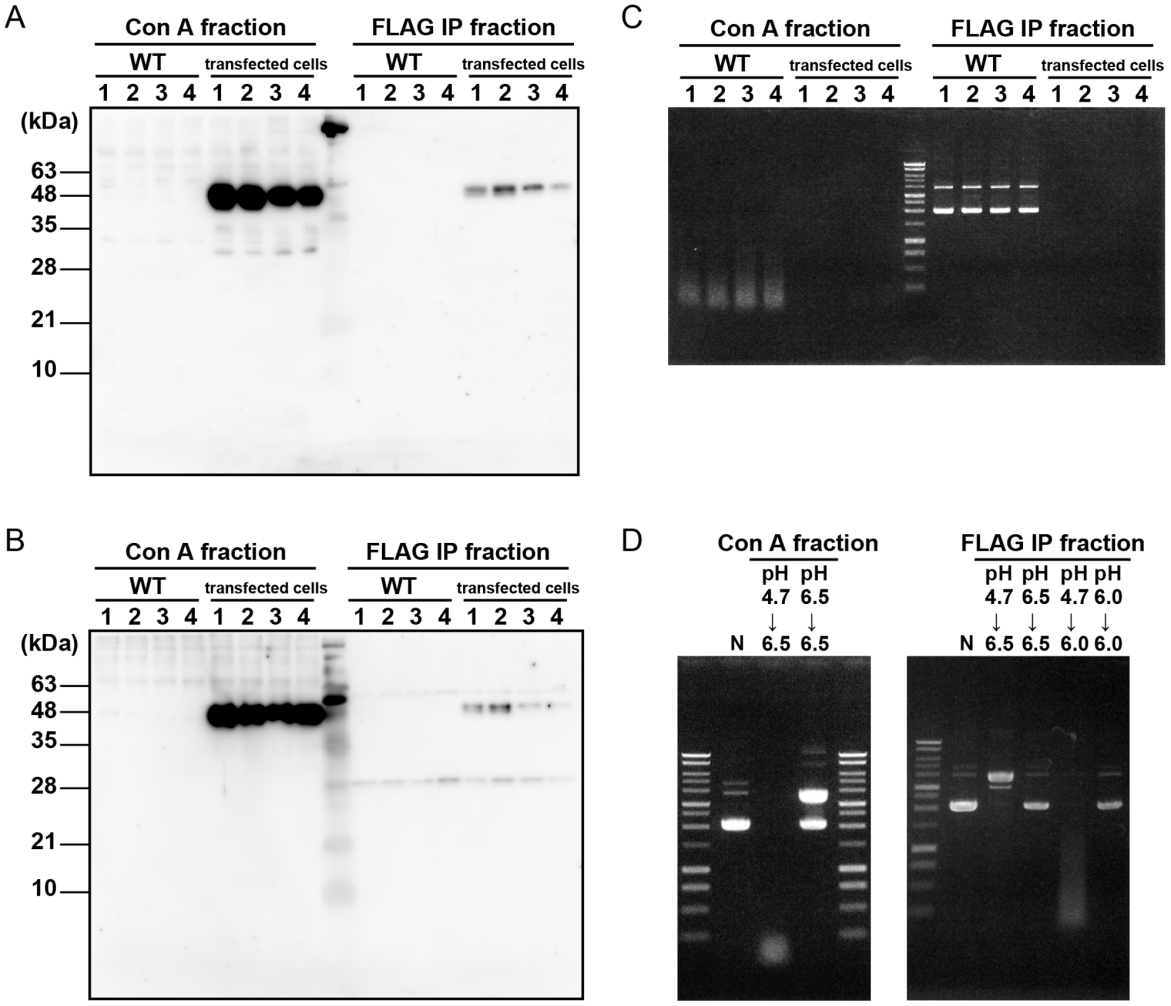
Secretion of DNase II and its DNase activity under acidic conditions. A, B, C, Extracellular DNase II purification from culture media of COS-1 cells or COS-1 cells stably expressing DNase II-FLAG-His (DNase II/COS-1) was performed as described in the experimental procedures. Con A fractions or anti-FLAG IP fractions were subjected to SDS-PAGE followed by Western blotting using anti-DNase II antibody (A), or anti-FLAG antibody (B). No DNase II forms were detected in the media that were obtained from non-transfected wild-type (WT) COS-1 cells, while intense bands immunoreactive to anti-DNase II or anti-FLAG antibody appeared at a molecular mass of 45 kDa from DNase II/COS-1 cells (A, B). DNase II assay was performed using each of the fractions (C). COS-1 cells or DNase II/COS-1 cells were cultured in DMEM (lane 1), with mannose-6-phosphate (M6P, lane 2), protease inhibitor cocktail (P.I., lane 3), or M6P and P.I. (lane 4). In transfected COS-1 cells, plasmid DNA was completely degraded at pH 4.7 in each lane applied (C). On the other hand, in WT COS-1 cells, plasmid DNA was weakly degraded in the Con A fractions at pH 4.7, but not in anti-FLAG IP fractions. D, DNase activity was measured after pre-incubation (see in experimental procedures). Plasmid DNA was significantly degraded when Con A fractions (left panel) or FLAG IP fraction (right panel) from transfected COS-1 cells were once pre-incubated at pH 4.7 for 2 hours and further incubated for 3 hours at pH 6.5 or pH6.0, respectively, whereas it was not degraded when the same fractions were pre-incubated only at pH 6.5. N: negative control experiment without a Con A fraction.

To evaluate the processing of DNase II under acidic conditions in lysosomes, the proform of DNase II was purified from the media, in which COS-I cells expressing a DNase II-FLAG were cultured either in the presence of mannose-6-phosphate (M6P) to prevent lysosomal enzymes from re-entering the cells by endocytosis, or in the presence of proteinase inhibitors to prevent degradation. The pro-DNase II was immunoprecipitated by anti-Flag antibody, while it was purified with Con A Sepharose, by which most of the lysosomal enzymes, including cathepsins secreted extracellularly, were co-purified. The purified proDNase II from transfected COS-I cells by Con A Sepharose or by immunoprecipitation with the anti-FLAG antibody demonstrated DNase activity in each lane applied when plasmid DNA was used as a substrate at pH 4.7, and the substrate was completely degraded (Figure 5C). In wild-type COS-1 cells no positive bands for DNase II were detected around molecular masses of 45 kDa in both western blotting using the anti-DNase II and anti-FLAG antibodies (Figure 5A and B). However, the purified culture medium by Con A Sepharose from wild-type COS-1 cells showed weak DNase activity at pH 4.7, but that by immunoprecipitation using anti-FLAG antibody from wild-type COS-1 cells did not. The result indicates that the Con A fractions from the culture medium of wild-type COS-1 cells contained proDNase II, although no protein bands for the proform were detected by western blotting (Figure 5C). We further examined whether proDNase II was activated under acidic conditions or not. The proDNase II purified from the Con A Sepharose fraction was pre-incubated at pH 4.7 or pH 6.5, followed by measuring DNase activity at pH 6.5. Pre-incubation at pH 6.5 was not enough to activate DNase II and the substrate was not degraded. However, proDNase II pre-incubated at pH 4.7 had significant DNase activity, and the substrate was degraded (Figure 5D). Moreover, we also examined DNase activity of proDNase II purified from the anti-FLAG IP fraction and found that it had DNase activity similar to that of proDNase II purified from the Con A Sepharose fraction (Figure 5D). It was also confirmed that pre-incubation of anti-FLAG immunoprecipitated proDNase II at pH 6.0 or pH 6.5 was unable to activate DNase II and the substrate was not degraded (Figure 5D). The results show that proDNase II in the neutral milieu has no DNase activity, but once proDNase II is exposed to acidic conditions together with or without cathepsins, proDNase II is activated and expresses DNase activity. Moreover, immunoprecipitated proDNase II from COS-I cells expressing DNase II-FLAG showed DNase activity at an acidic pH without proteolytic processing, indicating that the active site may be accessible by changing the conformation under acidic conditions (Figure 5C and D).

## Discussion

DNase II activity is recovered in a fraction equivalent to the content of cathepsin D and acid phosphatase [13]. Likewise, the *in vivo* roles of DNase II have been identified using knockout mice [6], [7], [9], [11]. However, the present study is the first to investigate the distribution and subcellular localization of the protein. In this study, we produced a polyclonal antibody against DNase II that was subsequently used for both Western blotting and immunocytochemical analyses.

During embryonic development, erythropoiesis takes place in the fetal liver and spleen. As development proceeds, the bone marrow is responsible for red blood cell production. Accumulation of undigested DNA was observed in the macrophages of the liver, spleen and bone marrow of both *DNase II^−/−^* and *DNase II^−/−^ IFN-IR^−/−^* mice [7], [11]. In the present study, immunocytochemistry was used to demonstrate that the DNase II protein is present in the lysosomes of both splenic macrophages and mononuclear cells in the bone marrow (Figure 2A–T). In particular, immunoelectron microscopy clearly showed that DNase II was present in CD68- and lamp1-positive lysosomes of macrophages (Figure 2U–W). These data are reasonable to explain the phenotypes of both *DNase II^−/−^* and *DNase II^−/−^ IFN-IR^−/−^* mice.

Lysosomal enzymes such as cathepsins B, L, and D are proteolytically processed during maturation. These enzymes are synthesized as preproproteins in the rough ER. The signal peptides are cotranslationally removed, and then the proenzymes are processed into enzymatically active single-chain forms, which undergo additional processing into heavy chain and light chain forms [35], [36]. There are two hypotheses regarding maturation of the DNase II protein. Biochemical purification of porcine DNase II indicated that the enzyme is composed of two or more (α1, α2 and β) subunits [13], [22], [23], [37]. However, it has been argued that these subunits are artificially generated during purification and the protein is active as a single polypeptide. This hypothesis is based on the observation that the endogenous DNase II protein produced by various human cell lines and the human DNase II overexpressed in mouse fibroblasts are detected as one band at 45 kDa [18], [19]. In the present study, the antibody to DNase II detected three protein bands in cultured cells overexpressing DNase II (Figure 1A). In addition to the 45-kDa protein, we observed two other unexpected protein bands of DNase II at molecular masses of 30 and 23 kDa. Of interest, these two proteins corresponded well with the endogenous DNase II proteins that appeared at 30 and 23 kDa (Figure 1B). Moreover, subcellular fractionation showed that the 45-kDa form was present in the microsomal fraction and that the 30- and 23-kDa forms were detected in lysosomes (Figure 3). Thus, mouse DNase II appears to be processed and activated during transport to lysosomes *in vivo*. Processing of lysosomal proteinases from the single-chain form to the heavy- and light-chain forms is inhibited by a cysteine protease inhibitor, E-64d [38]. Our data suggested that the behavior of DNase II is similar to that of lysosomal proteinases. Therefore, it seems likely that the 45-, 30-, and 23-kDa proteins are the pro-, single-chain, and heavy-chain forms of DNase II, respectively.

The present study showed that conversion of DNase II from the 30-kDa form to the 23-kDa form was largely inhibited in COS-1 cells expressing DNase II-FLAG-His by E-64d or a proteinase inhibitor cocktail, but not by pepstatin A, an aspartic proteinase inhibitor. The results indicate that cysteine proteinases play a key role in the processing of DNase II from the 30-kDa form to the 23-kDa form. However, since there is a possibility that pepstatin A does not work properly within the cells, the role of aspartic proteinases may not be rule out in the processing of DNase II. To further confirm the participation of cysteine proteinases, molecular forms of DNase II were examined using samples from the liver and spleen of mice deficient in lysosomal cathepsin B, L or D. Our data showed that processing from the 30-kDa form to the 23-kDa form is dependent, at least in part, on cathepsin L, a cysteine proteinase in the liver. It was interesting that the processing of DNase II was affected by proteinases in the liver but not in the spleen. This may be attributed to the fact that the activity of cathepsin L is higher in the liver than in the spleen [28], or there could be other proteinase(s) that is responsible for the processing of DNase II. It remains to be determined whether the processing of DNase II is directly executed by cathepsin L, the activity of DNase II in cathepsin-deficient mice are affected, and the cleavage sites in the DNase II molecule are unknown. The processing of porcine DNase II from the 46.5-kDa protein to the 35-kDa form is inhibited by chloroquine. This result suggests that lysosomal proteinases are involved in the processing [24]. In our experiment, however, the processing of mouse DNase II from the 45-kDa form to the 30-kDa form was not inhibited by the proteinase inhibitor cocktail (Figure 4A), but the activation of proDNase II occurred under pH 4.7 together with a Con A Sepharose fraction that included lysosomal enzymes or with an anti-DNase II IP fraction (Figure 5D). The result indicates that the activation of DNase II occurs at least in a pH dependent manner. Taken together, these lines of evidence suggest that the processing of the DNase II protein from the 45-kDa form to the 30-kDa form is largely dependent on the acidic milieu of the lysosome and further proteolytically processed in the lysosome.

The proposed α2 subunit of porcine splenic DNase II, which is the fragment closest to the carboxyl-terminal, starts from Ser110 and ends with Lys351 [23]. In murine DNase II, the amino acid residues that correspond to these sites are Ser110 and Glu351 of mouse DNase II, respectively. The calculated molecular mass of the sequence (from Ser110 to Glu351) of mouse DNase II is 27 kDa. However, taking into account the two possible glycosylation sites at Asn214 and Asn268, the actual molecular mass of the mouse DNase II protein may be equivalent to that of the α2 subunit of the porcine splenic DNase II protein. We could not confirm the presence of the small 10-kDa β subunit [22], [23], which might not have included the antigenic portion that is recognized by the antibody we used.

Similar to a previous report concerning HEK293 cells [39], the anti-FLAG antibody detected a protein band at 45 kDa in 293FT cells that overexpressed DNase II with the FLAG-His-tag (Figure 1A). When the antibody to mouse DNase II was used in Western blotting, we detected 45-, 30-, and 23-kDa proteins. However, the smaller two protein bands were not detected by the anti-FLAG antibody. This result suggests that mouse DNase II is processed proximal to the carboxyl terminus, as is cathepsin B [40]. In fact, 13 amino acids at the carboxyl-terminus of porcine DNase II are removed by processing [23]. However, we cannot exclude the possibility that only the attached tag was processed.

To the best of our knowledge, this study is the first to produce an anti-DNase II antibody which recognize the endogenous processed forms of DNase II and to use such an antibody to detect endogenous DNase II in the lysosomes of murine macrophages/mononuclear cells. Moreover, endogenous DNase II was processed to generate two proteins with the molecular masses of 30 kDa and 23 kDa, while cathepsin L was involved in the processing from the 30-kDa form to the 23-kDa form in the liver, but not in the spleen. Thus, the present data suggest that DNase II plays a pivotal role in the degradation of ingested DNA in the lysosomes of phagocytes.
